# Simple and robust high-throughput serum proteomics workflow with low-microflow LC–MS/MS

**DOI:** 10.1007/s00216-024-05603-3

**Published:** 2024-10-18

**Authors:** Yoondam Seo, Inseon Kang, Hyeon-Jeong Lee, Jiin Hwang, Soo Heon Kwak, Min-Kyu Oh, Hyunbeom Lee, Hophil Min

**Affiliations:** 1 Present Address: Doping Control Center, Korea Institute of Science and Technology (KIST), Hwarang-Ro 14-Gil 5, Seongbuk-Gu, Seoul, 02792 Republic of Korea; 2 Department of Chemical and Biological Engineering, Korea University, Seoul, 02841 Republic of Korea; 3 Department of Internal Medicine, Seoul National University Hospital and Seoul National University College of Medicine, Seoul, 03080 Republic of Korea; 4 Center for Advanced Biomolecular Recognition, Korea Institute of Science and Technology (KIST), Hwarang-Ro 14-Gil 5, Seongbuk-Gu, Seoul, 02792 Republic of Korea; 5 Department of HY-KIST Bio-Convergence, Hanyang University, Seoul, 04763 Republic of Korea; 6 Divison of Bio-Medical Science & Technology, KIST School, University of Science and Technology, Seoul, 02792 Republic of Korea

**Keywords:** High-throughput, Data-independent acquisition, Chronic kidney disease, Low-microflow

## Abstract

Clinical proteomics has substantially advanced in identifying and quantifying proteins from biofluids, such as blood, contributing to the discovery of biomarkers. The throughput and reproducibility of serum proteomics for large-scale clinical sample analyses require improvements. High-throughput analysis typically relies on automated equipment, which can be costly and has limited accessibility. In this study, we present a rapid, high-throughput workflow low-microflow LC–MS/MS method without automation. This workflow was optimized to minimize the preparation time and costs by omitting the depletion and desalting steps. The developed method was applied to data-independent acquisition (DIA) analysis of 235 samples, and it consistently yielded approximately 6000 peptides and 600 protein groups, including 33 FDA-approved biomarkers. Our results demonstrate that an 18-min DIA high-throughput workflow, assessed through intermittently collected quality control samples, ensures reproducibility and stability even with 2 µL of serum. It was successfully used to analyze serum samples from patients with diabetes having chronic kidney disease (CKD), and could identify five dysregulated proteins across various CKD stages.

**Supplementary Information:**

The online version contains supplementary material available at 10.1007/s00216-024-05603-3.

## Introduction

Clinical proteomics has substantially advanced, particularly in the identification and quantification of proteins in tissues or cells [1]. Tissue- and cell-based proteomics have made substantial progress in identifying and quantifying proteins with high precision and sensitivity, paving the way for more targeted and effective therapeutic interventions [2]. Despite these advances, blood remains a critical sample for biomarker discovery due to its reflection of the physiological and pathological states of the body [3, 4]. Blood proteomics is essential for identifying biomarkers that indicate disease presence, progression, and treatment response, thereby facilitating early diagnosis and personalized treatment strategies [5]. However, the high abundance of certain proteins, such as albumin and immunoglobulins, in blood complicates mass spectrometry (MS) analyses by masking the presence of clinically significant and less abundant proteins [6]. To address this issue, immunodepletion or affinity-based techniques can be employed to remove highly abundant proteins. Consequently, the overall workflow can take approximately 24 h, making it challenging to analyze large-scale cohorts within a reasonable timeframe, which limits its utility in clinical research and biomarker validation [7]. Large-scale studies on proteins are essential for understanding disease mechanisms and identifying therapeutic targets and biomarkers. A stable, economical, and high-throughput methodology is anticipated to be a key driver in advancing proteomics applications [8]. Therefore, the need for a high-throughput methodology, including sample preparation and liquid chromatography (LC)-MS methods, will become increasingly important.

However, traditional proteomics research predominantly employs nano-flow LC–MS/MS to continuously separate thousands of proteins from complex mixtures [9]. Although nano-flow LC provides high separation efficiency and sensitivity, its lengthy analysis time poses substantial limitations for large-scale studies. To address these limitations, Mann recently introduced a novel LC system called Evosep One. This system integrates directly with the stage tips, dramatically reducing the analysis time [10]. By increasing the flow rate to the 10–50 microflow range, the number of samples analyzed per day can reach up to 300 [11]. Advances in mass spectrometry, such as Bruker trapped ion mobility spectrometry-time of flight mass spectrometry (TIMS-TOF MS) and Thermo Astral instruments, support these high-throughput capabilities owing to their rapid analysis speeds. Mi et al. used a data-dependent acquisition mode (DDA-PASEF) with an 11.5-min runtime, employing Evosep and TIMS-TOF Pro, to identify 269 proteins [12, 13]. They showed that using a microflow LC/MS system makes the discovery of proteins feasible.

Despite these technological advancements, a substantial investment in facilities is required, which challenges many proteomic groups. Optimization of conditions using traditional instruments to achieve a yield similar to that achieved with the latest equipment can address this challenge. Using a budget-friendly system in a previous study, such as ultimate 3000 nano-LC and the Orbitrap Exploris 240, more than 300 protein groups were quantified with a 60-min gradient at a flow rate of 50 µL/min [14]. However, the capacity to analyze only 24 samples per day (SPD) requires a long analysis time for large-scale cohort studies.

In this study, we aimed to introduce a high-throughput clinical proteomics workflow with a short gradient using a low-microflow column based on a traditional LC–MS/MS system. To achieve this, conditions were optimized with a 150-µm internal diameter column operating at a flow rate of 1000 nL/min, balancing between sensitivity and a throughput of 80 SPD in data-independent acquisition (DIA). We optimized the critical parameters affecting protein identification, including the wide-window *m/z* range, resolution, and isolation window, to enhance the accuracy and comprehensiveness of protein quantification. In addition, we proposed a comprehensive high-throughput proteomics workflow that integrates a simplified sample preparation platform with DIA using a nano-LC–MS/MS system. The workflow was evaluated for reproducibility and robustness. Additionally, it was applied for patients with diabetes having chronic kidney disease (CKD) to identify CKD-related biomarkers. The integration of DIA improves the quantitative accuracy and proteome coverage, making this approach a valuable and cost-effective alternative for large-scale serum analysis.

## Materials and methods

### Reagents and chemicals

Formic acid (FA) was from Wako Pure Chemicals (Osaka, Japan). 0.1% FA in acetonitrile (ACN) and 0.1% FA in water were purchased from Thermo Fisher Scientific (Waltham, MA, USA). Triethylammonium bicarbonate (TEAB), Tris-(2-carboxyethyl) phosphine (TCEP), and 2-chloroacetamide (CAA) were obtained from Sigma-Aldrich (St. Louis, MO, USA). Indexed retention time (iRT) was sourced from Biognosys (Schlieren, Switzerland). Sequencing-grade modified trypsin was purchased from Promega (Madison, WI, USA).

### Ethics statement and sample collection

This study was approved by the Institutional Review Board of the Seoul National University Hospital (SNUH) (approval number: 2303–170-1417). In this study, we used serum samples from patients with diabetes at SNUH. Diagnosis of diabetes was investigated from 2016 to 2023. Informed consent was obtained from all participants. Patients with diabetes were classified into CKD stages based on clinical information, with stages 3 and 4 grouped together, resulting in three groups: stage 1, stage 2, and combined stages 3/4.

### Sample preparation for constructing a peptide spectral library

In proteomics research, a depletion procedure is utilized to simplify sample complexity. This procedure was conducted using 60 μL of pooled serum, following the Thermo Scientific Top 12 Abundant Protein Depletion Column protocol. The depletion spin column was first equilibrated at around 25 °C, after which 60 μL of serum was added. To ensure thorough mixing of the resin, the sample was vortexed and incubated at room temperature for 20 min. Post-incubation, the sample was eluted into a 2-mL collection tube via centrifugation at 1000 × *g* for 2 min. For enrichment, the eluted samples were filtered through Amicon Ultracel-3 K filters. Protein quantification was performed using the bicinchoninic acid assay, and 200 μg of protein was transferred to the Suspension TRAP (sTRAP) filter. All trapping, washing, and digestion steps were executed according to the Protifi S-TRAP protocol. The initial samples were divided into two aliquots of 30 and 50 μL, respectively, based on the loading capacity of the S-TRAP filter. Adjustments to the volumes of lysis buffer, reducing, alkylating, acidification, and binding/wash buffer were made according to the original sample volume, as per the protocol. The two aliquots were pooled, and 200 μg of peptides underwent high-pH fractionation. The peptides were dissolved in 100 μL of a loading solution containing 15 mM ammonium formate (pH 10) and 2% ACN for STAGE Tip fractionation. The separation of peptides was conducted using pipette-based RP microcolumns, which were prepared by packing a 200-μL pipette tip with Reprosil-Pur Basic C18 matrix and C18 Empore disk membranes at the base. After conditioning and equilibrating the microcolumns, peptides were loaded at pH 10, and 48 fractions were eluted using ACN step gradients in buffer solutions at pH 10. These fractions were subsequently combined into six fractions, dried using a Speedvac, and stored at − 20 °C until LC–MS/MS analysis.

### MS method for constructing a peptide spectral library

In this study, the six fractionated samples were analyzed using both data-dependent acquisition (DDA) and gas-phase fractionation data-independent acquisition (GPF-DIA). In DDA mode, a full scan ranging from 400 to 1000 m*/z* was acquired, with a mass resolution of 120,000, followed by data-dependent MS/MS, with a mass resolution of 15,000. The DDA analysis conditions were as follows: a full MS AGC target of 3e6, MS/MS AGC target of 1e5, dynamic exclusion of 15 s, mass isolation window of 1.6 m*/z*, minimum intensity threshold of 8e4, maximum injection time of 25 ms, and higher energy collisional dissociation (HCD) fragmentation with a normalized collision energy (NCE) of 30%.

In GPF-DIA mode, five injections were performed to analyze the following ranges: 500–550, 550–600, 600–650, 650–700, and 700–750 m*/z*. Each acquisition consisted of a survey scan at a mass resolution of 60,000, with a maximum injection time of 80 ms and an AGC target of 300%. MS/MS scans were conducted at a resolution of 45,000, with auto injection time and an AGC target of 3000%. For MS/MS scans, each 50 m*/z* range was divided into 16 windows (3 ± 1 m*/z* per isolation window). Normalized HCD energy varied for MS2 fragmentation: stepped energies of 22%, 26%, 30%. The data type was centroid.

### Streamlined sample preparation using 96-well plates

A high-throughput proteomics workflow, utilizing a 96-well plate, was implemented in a series of stages. First, 2 µL of serum was added to 23 µL of denaturation buffer (8 M urea, 20 mM CAA, and 10 mM TCEP in 50 mM TEAB). This mixture was then incubated at 60 °C for 25 min. After incubation, the mixture was diluted with 175 µL of 50 mM TEAB to reduce the urea concentration to less than 1 M. Next, 50 µL of the diluted mixture was transferred to a second plate. Trypsin was added at a 1:50 enzyme-to-protein ratio. The samples were incubated at 47 °C for 4 h. After incubation, the samples were acidified with 10% FA. Sequentially, 20 µL of processed sample was transferred to the third plate. Then, 20 µL of iRT, diluted 320-fold with loading A consisting of 0.1% FA and 2% ACN in distilled water (DW), was added. The final plate (third plate) was centrifuged at 4000 × *g* for 10 min and placed directly into the LC–MS/MS autosampler.

### LC–MS/MS conditions

The analysis was performed using a DIONEX Ultimate 3000 RSLC nanosystem. Samples were injected onto a precolumn (PepMap C18, 300 µm × 5 mm; Thermo Fisher Scientific) and eluted through an analytical column of ES906 (PepMap C18, 150 µm × 15 cm; Thermo Fisher Scientific). The flow rate was set to 1000 nL/min. The mobile phase comprised of 0.1% FA and 2% ACN in DW as solvent A and 0.1% FA in 80% ACN as solvent B. The initial gradient composition (5% solvent B) was gradually increased to 12% solvent B over 1.5 min. Subsequently, the sample was separated using a gradient from 12 to 35% solvent B from 1.5 to 14.5 min, followed by a linear increase to 95% B at 15.1 min. This composition was maintained for 1.9 min before it was reduced to 5% B. A post-run equilibration was performed for 1 min.

The spectral data were acquired using a Thermo Fisher Scientific Orbitrap Exploris 240 mass spectrometer. For the DIA analysis, a full scan ranging from 500 to 750 m*/z* was performed at a mass resolution of 60,000, maximum injection time of 80 ms, and AGC target of 300%. MS/MS scans were conducted at a resolution of 45,000 with auto injection time and an AGC target of 3000%. The MS1 range was divided into 18 windows (14 ± 1 m/*z* per isolation window). Normalized HCD energy for MS2 fragmentation varied with stepped energies of 22%, 26%, and 30%. The MS/MS data type was centroid.

### MS data processing

Raw files were processed using the direct DIA + (Deep) workflow in Spectronaut (Version 18.3, Biognosys AG, Switzerland) with default search settings. A human FASTA file was downloaded from UniProt to generate a spectral-free library. An additional in-house library was specified to create a hybrid library to improve the depth of proteome coverage. Carbamidomethylation was designated as a fixed modification, whereas N-terminal acetylation and methionine oxidation were considered variable modifications. Trypsin was used as the cleavage enzyme, allowing for a maximum of two missed cleavages. Mass tolerance was set dynamically for both MS1 and MS2 levels, with a correction factor of 1. To ensure accuracy, a *Q* value threshold of 1% against mutated decoys was used to filter identifications, achieving a false discovery rate of 0.01 at both peptide and protein levels. Imputation was performed using background signal settings, and the data were normalized using the integrated cross-run normalization feature in Spectronaut. Batch effect correction and missing value imputation were carried out using the quality control (QC)-based robust LOESS signal correction (QC-RLSC) method in StatTarget (Version 2.0.0.0) [15].

### Statistical analysis

Statistical analysis was conducted on the normalized and batch-corrected data. The resulting data were subjected to principal component analysis (PCA) and partial least-squares discriminate analysis (PLS-DA) using SIMCA (Version 17.0). Linear discriminant analysis (LDA) was conducted using the R package MASS (version 7.3–60) to visualize the distribution of the three CKD stage groups. Additionally, we performed the Kruskal–Wallis test with Dunn’s post hoc analysis using the R package FSA (version 0.9.5) and calculated fold changes between the comparison groups. Finally, boxplots were generated with Origin 2022 (Version 9.9.0.220).

## Results and discussion

### High-throughput MS systematically analyzes the serum proteome at large scale

**Fig. 1:**
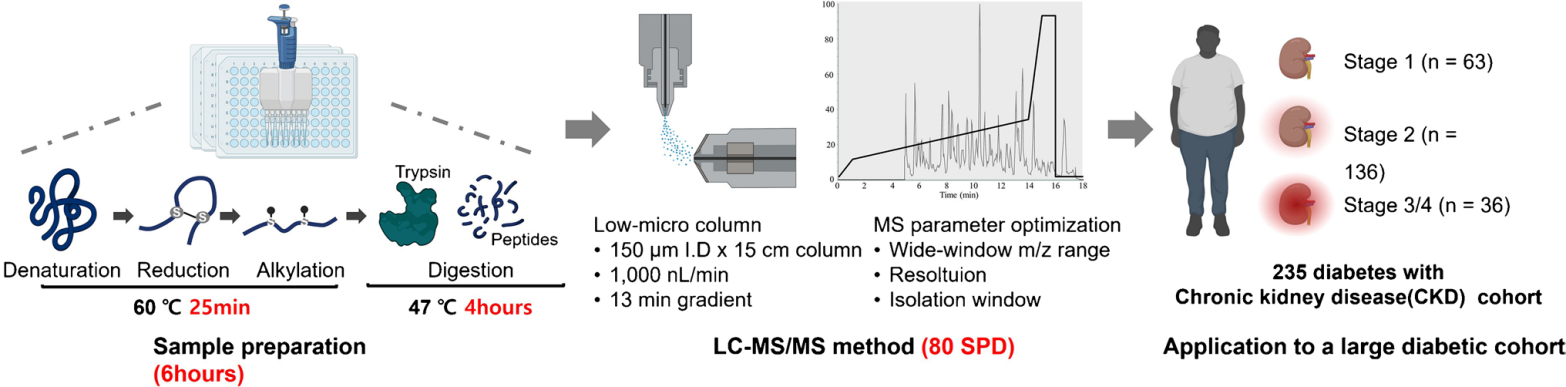
High-throughput proteomics analysis workflow from sample preparation and LC–MS/MS method to its application. The sample preparation, representing the under 6-h sample preparation, including denaturation, reduction, alkylation, and trypsin digestion. The LC–MS/MS method optimization with MS parameters using the low-microflow column is shown. The figure sequentially shows the application of the workflow to samples from 235 patients with diabetes having chronic kidney disease across different stages. SPD, samples per day

High-throughput proteomic techniques improve both the speed of analysis and accuracy and depth of proteome coverage [16]. Several quantitative proteomics studies have proposed multiplexed sample preparation using a plate format to increase the throughput [17]. Similarly, in this study, sample preparation was optimized using 96-well plates to minimize the processing steps. Briefly, this involved 25 min of simultaneous denaturation and reduction/alkylation, followed by 4 h of trypsin digestion, enabling the preparation of 96 samples within 6 h (Fig. 1). In the majority of proteomic analyses, trypsin is a standard protease, and the sample is typically incubated with it overnight at 37 °C. However, during large-scale sample preparation, the duration of proteolysis does not affect the reproducibility [18]. To maximize throughput and efficiency, we accelerated the process by raising the incubation temperature, which allowed us to select a 4-h trypsin digestion at 47 °C based on previous reports [19]. Previous studies have also shown that increasing the denaturation temperature and reducing the reaction time can enhance protein identification yields [14]. Therefore, we could save the denaturation time by preparing the denaturation buffer consisting of 8 M urea, TCEP, and CAA simultaneously and heating at 60 °C for 25 min [20]. As a result, direct analysis of the samples on the prepared plates using LC–MS/MS highlighted the need to optimize the high-throughput LC–MS/MS method for efficiency and proteome coverage.

For high-throughput proteomic analysis, two major data acquisition approaches can be used: DDA and DIA. Unlike DDA, which requires pre-fractionation for deep coverage, DIA offers improved reproducibility and higher proteome coverage in a single injection [21], making it an attractive alternative for high-throughput proteomics [22]. Additionally, nano-flow ultra-performance liquid chromatography, despite its sensitivity, was limited to a low-throughput technique, processing only 10–20 SPD [12]. To address the limitations of conventional analysis methods, we developed an 18-min DIA method using a low-microflow column in a traditional nano-LC/MS system capable of analyzing up to 80 SPD. We also optimized the MS acquisition parameters, including the wide-window range, resolution, and isolation window specific to the columns and instruments used to ensure reliable identification of a large number of proteins (Fig. 1). We then evaluated a workflow, including sample preparation completed within 6 h and the 18-min DIA method for profiling protein biomarkers. The workflow was assessed for its robustness and reproducibility. Subsequently, this workflow was applied to profile serum samples from a cohort of patients with diabetes having CKD to identify biomarkers that differ across CKD stages (Fig. 1). We identified proteins in patients with diabetes at various CKD stages, consistent with previously reported biomarkers of kidney disease. This demonstrated the potential applicability of our workflow in clinical research.

### Evaluation of the DIA approach for protein identification

**Fig. 2:**
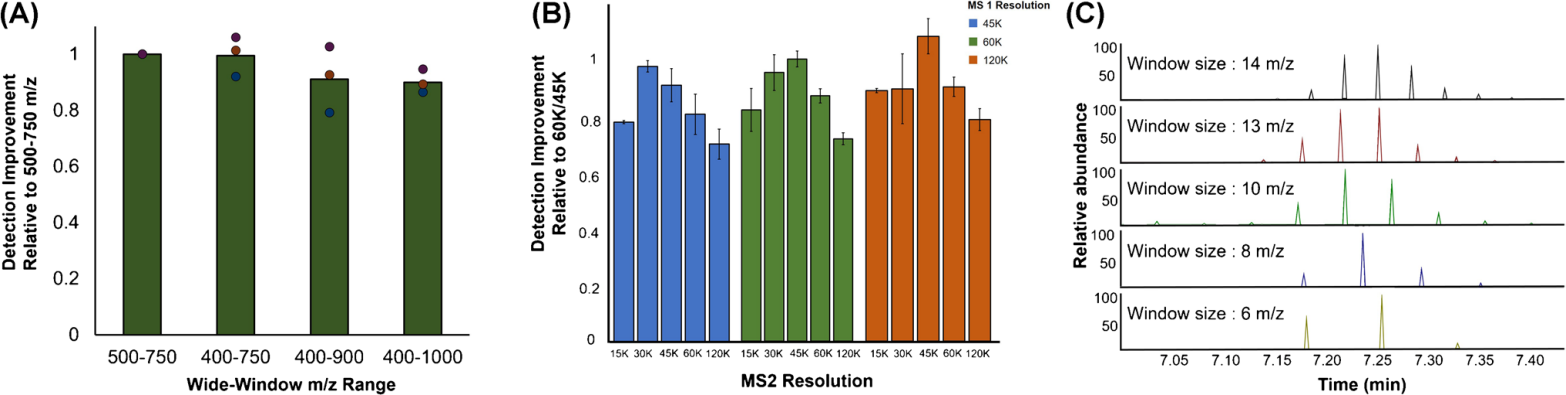
Optimization of the wide-window *m/z* range, resolution, and isolation window for the DIA method. **A** Comparison of the effect of four wide-window *m/z* ranges on the number of identified proteins. Green boxes representing the ratio of identified proteins compared with that for the 500–750 m*/z* range. Purple, brown, and blue circles corresponding to replicates 1, 2, and 3, respectively. **B** The effect of MS/MS resolving power on the number of proteins with fragmentation information across three different full MS resolution settings. Error bars representing the standard deviation of the number of identified proteins. **C** The number of data points as a function of MS/MS resolution. DIA, data-independent acquisition

The use of large-inner-diameter columns/emitters and microflow rates can increase the sample throughput. A balance between throughput and proteome depth is essential for large-scale profiling in LC–MS/MS using microflow rates. This was achieved by optimizing the LC and MS parameters for efficient peptide separation, precursor isolation, and fragmentation. These compromises manifest as a result of three competing objectives: (a) maximizing the number of identified proteins, (b) minimizing the coefficient value, and (c) maximizing the number of points measured across every chromatographic peak [23]. The acquisition parameters for DIA, including the wide-window *m/z* range, resolution, and isolation window, influence the number of precursors available for co-fragmentation [24]. We systematically tested each of these parameters and employed optimal values for the DIA analysis. Pooled serum tryptic samples were analyzed using four MS1 ranges. Given that most tryptic peptides were detected in the 400–900 m*/z* range in a previous study, we extended our tests to the surrounding range [23]. The number of identified proteins was compared based on the 500–750 m*/z* range under different conditions. We found that the maximum number of proteins was detected in the 500–750 m*/z* range because of its better window selectivity (Fig. 2a).

High resolution is essential for mass accuracy and selectivity, leading to an increased rate of protein identification. We compared the ratio and variance of identified proteins across 12 different conditions based on MS resolutions of 60 K and 120 K, combined with various MS/MS resolutions. Our analysis revealed that the combination of 120 K MS and 45 K MS/MS resolution resulted in the highest number of protein identifications, with an average increase of approximately 15% compared with that in the 60 K MS and 45 K MS/MS combination (Fig. 2b). However, considering that 120 K MS has a scan rate (Hz) less than half that of 60 K MS [25], we anticipated that this would result in fewer scan points for subsequent quantitative analysis. This is particularly critical for our method, in which peak widths are typically 8–9 s, making scan speed and cycle time crucial factors. For DIA-MS analysis, a balance must be achieved between resolution, isolation window width, and cycle time to obtain reliable results. While higher resolution can increase protein identification, it can also lead to fewer scan points, potentially compromising the quantification accuracy. We found that the 60 K MS and 45 K MS/MS combination had a low coefficient of variation (CV) of 4.1%, providing a better balance between identification and quantification capabilities.

Further optimization involved adjusting the isolation window width. We observed that wider isolation windows led to shorter cycle times (Table 1), resulting in more data points per peak for protein quantification (Fig. 2c). However, increasing the size of the isolation window while decreasing its number typically diminishes the precursor identification rates, illustrating the challenge of deconvoluting highly complex spectra using wide isolation windows.

**Table 1:** A comparison of the different data-independent acquisition isolation windows

Resolution		Window		Cycle time (s)
MS1	MS2	Size (*m/z*)	Number	
60 K	45 K	14	18	1.8
		13	20	1.8
		10	25	2.4
		8	31	3.0
		6	41	3.6

Considering these factors, we selected the 60 K MS and 45 K MS/MS combination for further analysis. We determined that the advantages gained from adjusting other parameters, such as the isolation window, could compensate for the slight difference in protein identifications while maintaining the benefits of faster acquisition at 60 K MS resolution. Consequently, we optimized the window size to 14 m*/z* to ensure sufficient data points per peak for our high-throughput DIA analysis.

### Application of high-throughput workflow for serum proteomic profiles in diabetes CKD stage subtypes

#### Evaluation of the quantitative analysis within the high-throughput workflow

**Fig. 3:**
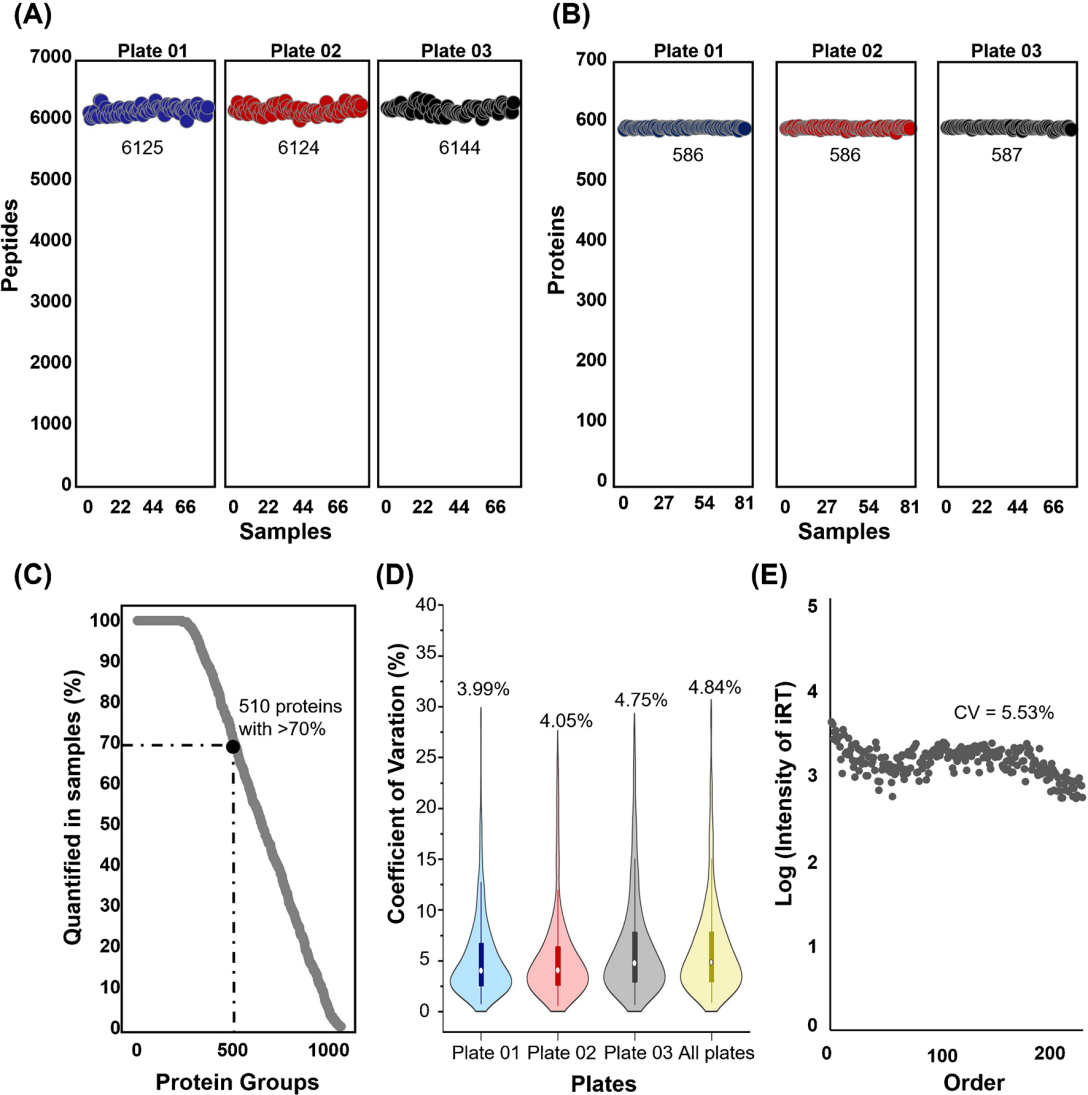
Performance evaluation of the 96-well plate sample preparation. **A** Number of peptides identified on each of the three plates using the 18-min method (80 SPD). **B** Number of proteins identified on each of the three plates using the 18-min gradient method (80 SPD). **C** Quantitative completeness across diabetes samples: 510 proteins with 70% completeness among 1027 proteins. **D** The coefficient of variation (CV) of the numbers of the identified proteins within and between the three plates, with the median CV. **E** Protein-level plots for iRT demonstrating stability of optimized workflow. iRT, indexed retention time; SPD, samples per day

A high-throughput workflow with streamlined sample preparation and an 18-min DIA approach (80 SPD) was applied to quantify proteins in 235 diabetic cohorts. The clinical information for the cohort is provided in Table 2. A total of 6684 peptides were identified in the raw data, and 589 proteins were quantified after correcting for the raw data using StatTarget. The quantification results were compared for each plate to determine differences within and across plates. This workflow consistently ensured the reproducible quantification of proteins. In the large-scale samples, we quantified approximately 6000 peptides and 590 proteins (Fig. 3a and b). The numbers of peptides and proteins were consistently detected across all three plates. This suggests that stable results can be obtained, even under varying sample conditions, thereby ensuring workflow reliability. In addition, consistent peptide and protein quantification across multiple plates highlighted the robustness of our method, demonstrating its ability to analyze large-sample cohorts with high reproducibility. To evaluate the robustness of the optimized workflow, we verified the completeness of the quantified proteins in the raw data. According to the BGS Factory Report in Spectronaut software, we were able to quantify a total of 1027 protein groups, with approximately 236 proteins quantified with 100% completeness and 510 proteins quantified with 70% completeness (Fig. 3c). The protein quantification within and across the three plates showed highly comparable coefficients of variation, consistently below 5% (Fig. 3d). For the stable analysis of samples, we measured the quality using spiked iRT, which was used as an internal standard for all the analyzed samples. This approach is consistent with previous studies highlighting the importance of iRT for calibration of retention time in large-scale cohort analyses, ensuring data consistency and reliability [26]. Therefore, the intensity of iRT spiked at a consistent concentration was confirmed using DIA analysis and the intensity remained stable with a CV% of 5.53%. The low CVs further emphasize the reliability of the quantification process. This workflow not only enhances throughput, but also maintains data quality, paving the way for effective biomarker discovery in large-scale cohorts.

**Table 2:** Clinical characteristics of the cohort

Parameters	CKD stages			
	Stage 1 (*n* = 63)	Stage 2 (*n* = 136)	Stage 3/4 (*n* = 36)	*p*-value
Gender (male:female)	22:41	78:58	16:20	-
Age (y)	59.17 ± 12.32	65.11 ± 9.84	67.97 ± 8.59	5.72E^−05^
ALP (IU/L)	73.9 ± 25.27	66.04 ± 18.39	74.03 ± 23.13	2.20E^−02^
ALT (IU/L)	30.54 ± 27.33	25.36 ± 16.87	21.86 ± 13.32	8.42E^−02^
AST (IU/L)	25.78 ± 13.94	25.46 ± 12.38	22.94 ± 11.09	5.15E^−01^
HbAlc (%)	7.5 ± 1.4	7.06 ± 0.89	7.36 ± 1.12	2.00E^−02^
TC (mg/dL)	175.15 ± 37.69	159.84 ± 34.29	170.03 ± 52.56	2.86E^−02^
HDL-C (mg/dL)	54.79 ± 17.01	51.26 ± 13.22	47.25 ± 11.31	3.67E^−02^
LDL-C (mg/dL)	93.58 ± 29.34	86.8 ± 29.11	87.62 ± 34.61	3.31E^−01^
eGFR (mL/min/1.73 m^2^)	105.69 ± 16.19	76.05 ± 8.56	43.64 ± 12.29	2.97E^−69^
Creatinine (mg/dL)	0.65 ± 0.11	0.91 ± 0.14	1.7 ± 1.13	3.13E^−22^
Total protein (g/dL)	7.25 ± 0.45	7.3 ± 0.42	7.31 ± 0.53	7.57E^−01^
FBG (mg/dL)	157.94 ± 48.4	135.1 ± 34.48	135.69 ± 28.4	3.40E^−04^

Data are presented as mean ± SD, unless otherwise indicated

*ALP *alkaline phosphatase, *ALT *alanine transaminase, *AST *aspartate aminotransferase, *HbA1c *hemoglobin A1c, *TC *total cholesterol, *HDL-C *high-density lipoprotein cholesterol, *LDL-C* low-density lipoprotein cholesterol, *eGFR* estimated glomerular filtration rate, *FBG* fasting blood glucose

#### Assessment of method reproducibility and biomarker discovery in serum proteomics

**Fig. 4:**
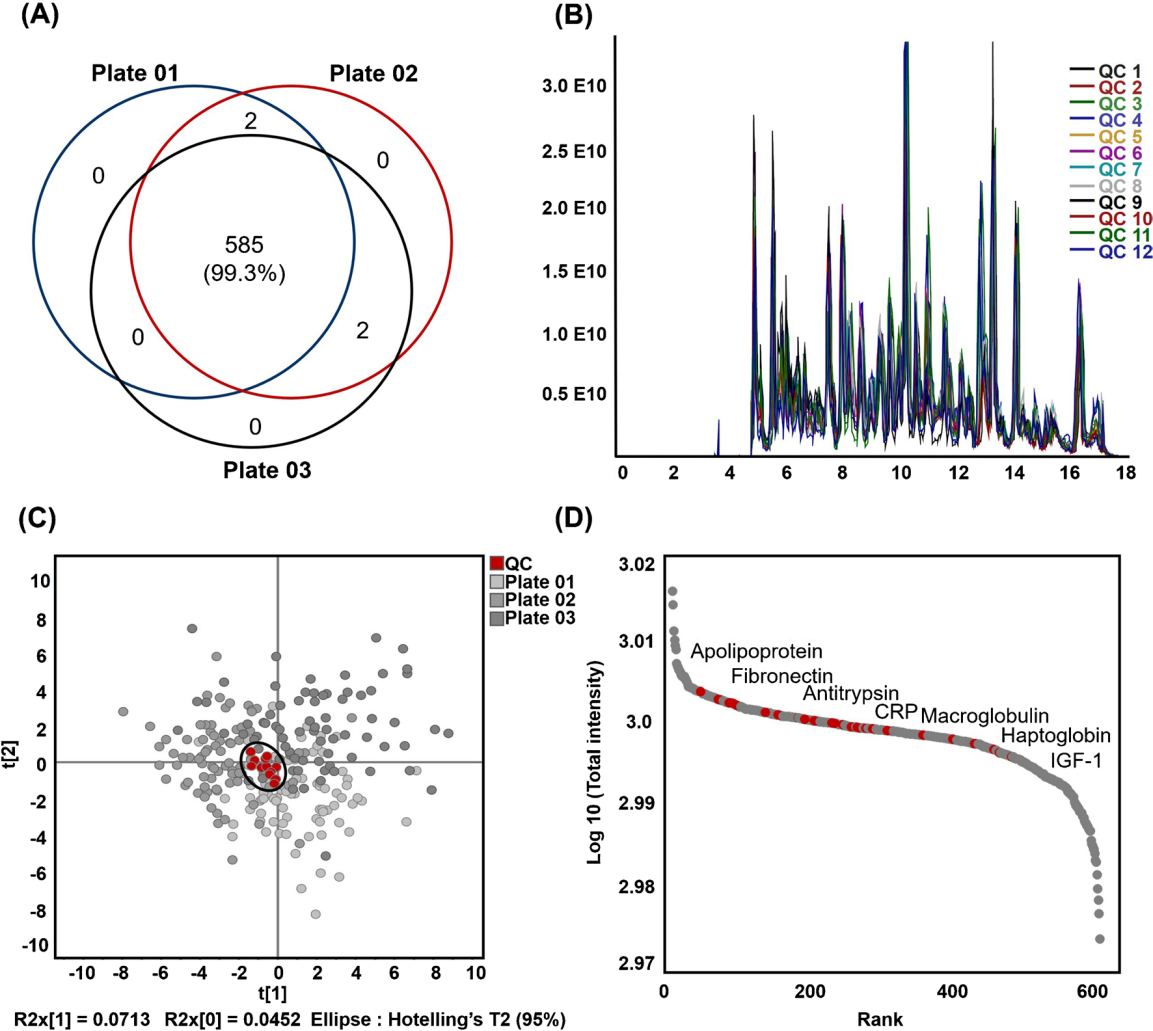
Reproducibility of proteomic data across triplicate analyses. **A** The overlap of identified proteins in three replicates using the same analytical method. **B** Overlapped base-peak total ion chromatograms for 12 QC samples. **C** PCA score plot of the study samples, including 235 individual serum and 12 QC samples. Red points indicate QC samples. **D** The dynamic range of the serum proteome, with 33 FDA-approved biomarkers marked in red. PCA, principal component analysis; QC, quality control

To evaluate the robustness and reproducibility of our proteomic workflow, which was capable of quantifying approximately 600 proteins, we assessed three independent QC samples from each plate. Pooled serum was used as the QC sample, and four QC samples were analyzed per plate. The Venn diagram in Fig. 4a illustrates the overlap of the identified proteins across the three QC samples from each plate. The large intersection area indicates that 99.3% of common proteins were identified in all three samples, demonstrating the robustness of our method for protein detection. The minimal variation observed among triplicate measurements ensured confidence in the accuracy and reliability of the proteomic data generated using this method. The overlapping chromatograms for all the QC samples exhibited excellent reproducibility (Fig. 4b). PCA, which is useful for assessing sample similarity, was employed to evaluate the proximity among QC samples [27]. After correcting for batch effects and filtering out missing values above 0.8 using the StatTarget software, PCA showed that the QCs clustered consistently in the center of the plot on two-component axes. In contrast, the individual samples were randomly distributed (Fig. 4c). The combined data from the Venn diagram and PCA plot provide clear evidence that our proteomic analysis method is highly reproducible. To assess the effectiveness of our workflow for identifying clinically relevant biomarkers, we cross-referenced our quantified protein list with FDA-approved biomarkers. Notably, 33 protein groups were identified among 109 FDA-approved protein biomarkers (Fig. 4d) [28]. Information regarding the protein groups observed in the QC samples is provided in Table S1. Notably, these 33 FDA-approved biomarker proteins were also consistently detected in all patient samples, demonstrating the robustness of our high-throughput method in identifying clinically relevant proteins. This enhanced capability is particularly valuable for studies on complex diseases, where analyzing a large number of samples is essential for obtaining substantial biological insights and identifying potential therapeutic targets.

#### Protein profiling and CKD-associated biomarkers in diabetes samples

CKD represents a significant public health challenge globally, characterized by its high prevalence and poor prognosis. Over 10% of adults show signs of declining kidney function, often without symptoms in the early stages. This asymptomatic nature underscores the importance of research into the different CKD stages and has prompted numerous studies employing diverse methodologies [29].

In this study, we employed a low microflow-based proteomics analysis method, optimized for high-throughput analysis, to examine protein expression across different CKD stages. Our objective was to validate the efficiency of the method in rapidly identifying differential protein expression and proposing candidate biomarkers pertinent to CKD stages.

**Fig. 5:**
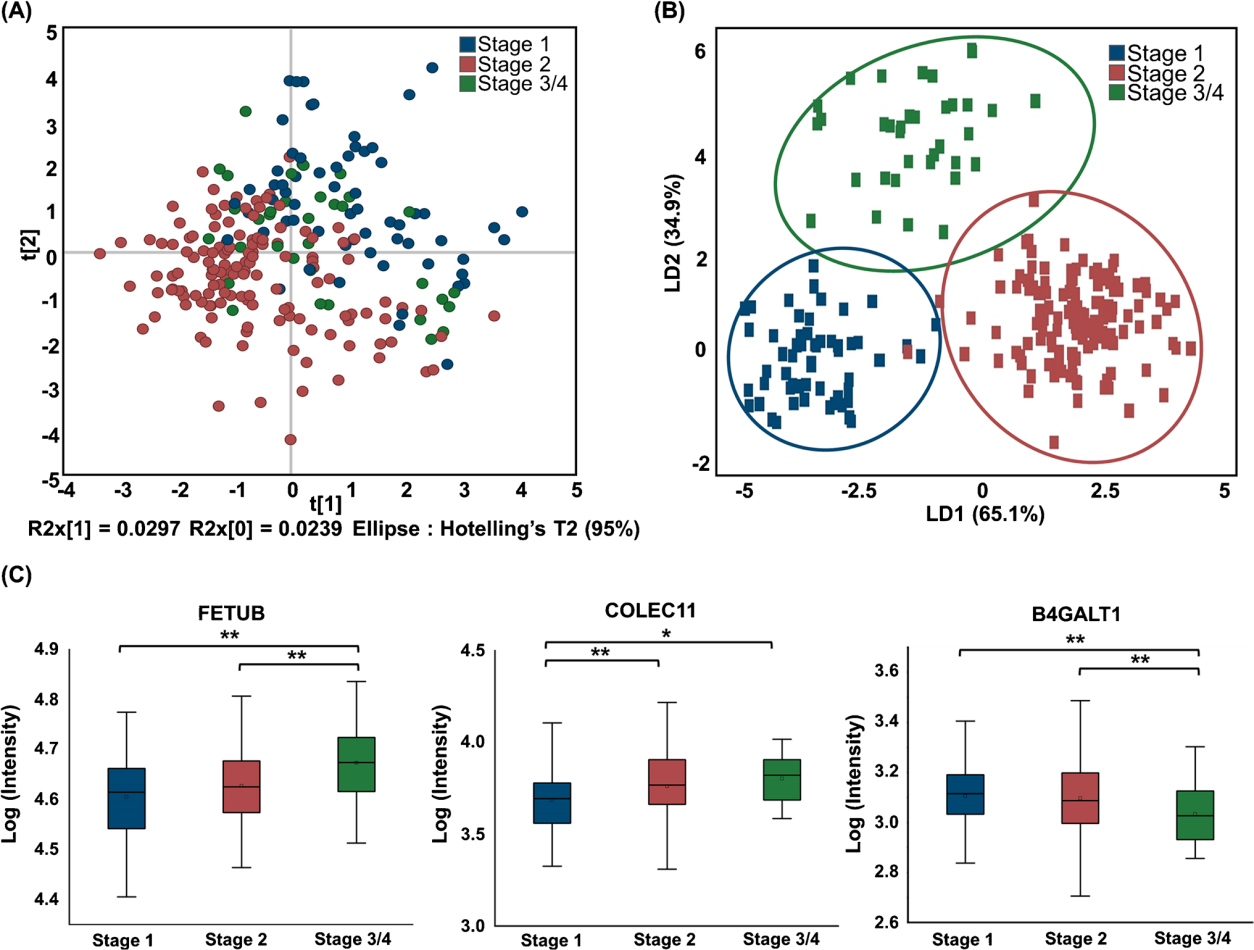
Application of the method to a clinical cohort of patients with diabetes having chronic kidney disease. **A** The PLS-DA score plots of serum proteomics data comparing the three CKD stages. **B** The LDA plot based on the top 197 proteins in PLS-DA plot and statistical analysis. **C** Three proteins with increased or decreased expression as CKD stage progresses; Kruskal–Wallis test with Dunn’s post hoc analysis; *p < 0.05, **p < 0.01. CKD, chronic kidney disease; LDA, linear discriminant analysis; PLS-DA, partial least-squares discriminate analysis

For a dataset comprising 589 quantifiable proteins, we employed PLS-DA and Kruskal–Wallis tests to analyze protein expression across CKD stages. PLS-DA was instrumental in differentiating CKD stages by identifying 186 key proteins with variable importance in projection (VIP) values exceeding 1, indicating their potential as biomarkers for CKD progression (Fig. 5a, Table S2). These proteins were further scrutinized using Kruskal–Wallis and Dunn’s tests, which are non-parametric methods suitable for addressing unequal sample sizes among groups. This analysis revealed 31 differentially expressed proteins (DEPs) with significant differences in expression. In particular, we identified 14 proteins with significant differences between stage 1 and stage 2, 20 proteins between stage 1 and stages 3/4, and 18 proteins between stage 2 and stages 3/4 (Table S3). Among these, proteins with a *p*-value less than 0.05 included 4 that were either upregulated by more than 1.2-fold or downregulated by less than 0.8-fold in the comparison between stage 1 and stage 2, 13 proteins in the comparison between stage 1 and stages 3/4, and 10 proteins in the comparison between stage 2 and stages 3/4. The integration of these datasets resulted in a refined list of 197 proteins, which were subjected to linear discriminant analysis (LDA). This analysis demonstrated effective classification of CKD stages based on the identified proteins, thereby enhancing our understanding of CKD by elucidating how these proteins differentiate between disease stages (Fig. 5b).

Among the 31 DEPs identified through our statistical analyses, three proteins—fetuin-B (FETUB), collectin-11 (COLEC11), and beta-1,4-galactosyltransferase 1 (B4GALT1)—were particularly noteworthy owing to their distinct expression patterns across CKD stages (Fig. 5c). FETUB and COLEC11 showed progressively increasing expression levels with advancing CKD stages, whereas B4GALT1 exhibited a decreasing trend.

Previous studies have elucidated the roles of these proteins in CKD. FETUB a member of the cystatin superfamily of cysteine protease inhibitors, plays a crucial role in the pathogenesis of metabolic diseases [30]. Elevated FETUB levels have been linked to enhanced insulin resistance, potentially contributing to CKD progression. Our findings of increased FETUB expression across CKD stages corroborate its potential as a biomarker for disease progression [31].

COLEC11, a pattern recognition molecule in the innate immune system, is primarily expressed in the adrenal gland, liver, and kidney. It plays a critical role in activating the innate defense system by recognizing pathogen-associated molecular patterns, initiating complement activation, and promoting phagocytosis [32]. The observed increase in COLEC11 expression with CKD progression suggests a compensatory mechanism to address heightened infection susceptibility in CKD patients. Elevated COLEC11 levels may also serve as biomarkers for disseminated intravascular coagulation, a severe complication in advanced kidney disease [33].

B4GALT1, an enzyme involved in glycosylation processes, is crucial for cell–cell and cell–matrix interactions, essential for maintaining the kidney structure and function [34]. Its significance has been noted in studies identifying biomarkers for kidney disease associated with early-onset type 2 diabetes. The decreasing expression of B4GALT1 observed in our study may indicate disruptions in normal cellular interactions and glycosylation patterns, contributing to kidney deterioration [35].

The differential expression patterns of these proteins, as revealed by our high-throughput proteomic analysis, offer valuable insights into the molecular mechanisms underlying CKD progression. These findings not only underscore the potential of these proteins as biomarkers but also highlight the complex interplay between metabolic regulation, immune function, and structural integrity in CKD pathogenesis. Further research is necessary to elucidate the precise mechanisms by which these proteins influence disease progression and to explore their potential as therapeutic targets in CKD management. The results obtained using our high-throughput method, consistent with those of previous studies on kidney diseases, suggest its suitability for large-cohort studies. Future research should aim to validate these findings in larger, diverse populations and to investigate the mechanistic roles of these proteins in CKD progression.

## Conclusion

In this study, we developed a high-throughput workflow that enables efficient and reproducible protein analysis without the need for automated equipment. The streamlined 96-well plate workflow significantly reduces sample preparation time to under 6 h. Additionally, an 18-min DIA approach was employed with a nano-LC/MS system. This system utilizes a low-microflow column with a 1000 nL/min flow rate and a short 18-min analysis. Key parameters, including the wide-window *m/z* range, resolution, and isolation window values, were also optimized to maximize the identification of protein groups within serum samples. This workflow allowed the analysis of 80 SPD, highlighting efficiency and suitability for large-scale studies. The evaluation of reproducibility and robustness demonstrated consistent protein identification and stable QC sample chromatograms. Thirty-three FDA-approved serum markers were identified, suggesting that this workflow is suitable for the detection and analysis of large-scale clinical serum samples. Consequently, the developed workflow offers a cost-effective, time-efficient, and highly reproducible approach for large-scale clinical proteomics. It can be implemented without automation equipment or additional instruments, making it highly accessible to proteomic research laboratories. Although this optimized workflow can be generally applied to traditional nano-flow LC/MS systems, additional optimization is required to apply it in other conditions. When applied to a diabetes cohort with CKD, we identified a total of 589 proteins, with significant findings related to the expression of five proteins that varied with CKD stages. As clinical information was not controlled in this study, we cannot account for the variations in protein expression due to this factor. Future research incorporating clinical information is required to identify more reliable biomarkers associated with kidney disease in patients with diabetes.

## Supplementary Information

Below is the link to the electronic supplementary material.

Supplementary file1 (XLSX 30 KB)
